# Association of Aβ monomers with cerebral amyloid angiopathy in brains without parenchymal Aβ deposition

**DOI:** 10.1093/braincomms/fcag051

**Published:** 2026-02-19

**Authors:** Lei Liu, Lei Yu, Vladislav A Petyuk, Alifiya Kapasi, Han Bin Yoo, Adriana Saba, Hyun-Sik Yang, Jasmeer P Chhatwal, David A Bennett

**Affiliations:** Brigham and Women’s Hospital, Harvard Medical School, Boston, MA 02139, USA; Rush Alzheimer’s Disease Center, Rush University Medical Center, Chicago, IL 60612, USA; Biological Sciences Division, Pacific Northwest National Laboratory, Richland, WA 99354, USA; Rush Alzheimer’s Disease Center, Rush University Medical Center, Chicago, IL 60612, USA; Brigham and Women’s Hospital, Harvard Medical School, Boston, MA 02139, USA; Brigham and Women’s Hospital, Harvard Medical School, Boston, MA 02139, USA; Brigham and Women’s Hospital, Harvard Medical School, Boston, MA 02139, USA; Brigham and Women’s Hospital, Harvard Medical School, Boston, MA 02139, USA; Rush Alzheimer’s Disease Center, Rush University Medical Center, Chicago, IL 60612, USA

**Keywords:** soluble Aβ, cerebral amyloid angiopathy (CAA), mass spectrometry, Alzheimer’s disease, Aβ monomers

## Abstract

β-Amyloid (Aβ) deposition is a hallmark of both Alzheimer’s disease and cerebral amyloid angiopathy. Whilst insoluble Aβ aggregates have been extensively studied, the role of soluble Aβ monomers in vascular amyloid pathology—and their association with cognitive decline—remains unclear in plaque-free brains. This study examined whether soluble cortical Aβ species are associated with cognitive outcomes and amyloid-related pathologies, including cerebral amyloid angiopathy, in the absence of parenchymal Aβ deposition. We examined post-mortem cortical tissue from nearly 200 individuals without parenchymal Aβ deposition, drawn from two longitudinal community-based cohorts. Soluble Aβ37, Aβ40 and Aβ42 were quantified by immunoassays, and total Aβ levels were measured using selected reaction monitoring proteomics. Associations with semiquantitative cerebral amyloid angiopathy burden and longitudinal cognitive trajectories were assessed using regression models adjusting for age, sex and education. Higher levels of soluble Aβ—particularly longer species such as Aβ42, reflected by elevated Aβ42/40 and reduced Aβ37/42 ratios—were significantly associated with greater cerebral amyloid angiopathy severity. Whilst immunoassay based total Aβ and Aβ ratio measures showed limited associations with cognitive outcomes, total Aβ levels quantified by selected reaction monitoring remained significantly associated with global cognitive decline. These findings support a pathogenic role for certain soluble Aβ monomers in vascular amyloid deposition. In contrast, cognitive impairment may be driven by other amyloid species such as oligomeric or extended Aβ forms. Aβ ratios may serve as specific markers for cerebral amyloid angiopathy and provide insights into early therapeutic strategies targeting vascular amyloid pathology.

## Teaser

Liu *et al*. report that soluble Aβ monomers, particularly longer species such as Aβ42, are strongly associated with cerebral amyloid angiopathy in plaque-free human brains, whereas cognitive decline relates more to aggregated or extended Aβ forms. These findings highlight soluble Aβ ratios as potential early markers of vascular amyloid pathology.

## Graphical Abstract

**Graphical Abstract:**
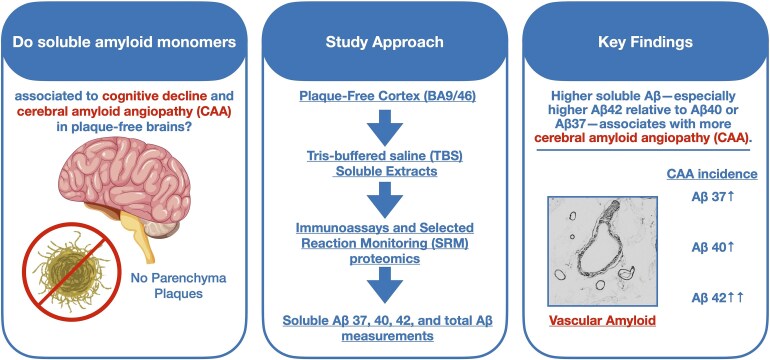

## Introduction

Alzheimer’s disease (AD) and cerebral amyloid angiopathy (CAA) are two of the most common age-associated neuropathological conditions, both characterized by the accumulation of β-amyloid (Aβ) proteins.^1,2^ Aβ pathology has long been recognized as a central feature of AD, with deposition into insoluble plaques regarded as a key hallmark. However, a growing body of evidence suggests that soluble Aβ species, such as oligomers,^3-6^ may also play critical roles in disease mechanisms by inducing synaptic dysfunction and neurotoxicity. Whilst substantial progress has been made in understanding Aβ plaque deposition, the clinical and pathological significance of soluble Aβ monomers in the absence of insoluble deposits remains poorly understood.

In our previous work,^7^ we demonstrated that soluble Aβ proteins, quantified using selected reaction monitoring (SRM) proteomics,^8^ were associated with cognitive decline even in individuals without insoluble Aβ deposition. This observation highlighted the importance of soluble Aβ in driving neurodegeneration and cognitive impairment. Importantly, we identified that Aβ was also linked to CAA, suggesting a vascular contribution to Aβ-related pathology. However, the SRM approach lacks specificity for individual Aβ species and cannot differentiate between monomers, oligomers and longer Aβ fragments, complicating result interpretation. The tryptic peptide used for Aβ quantification (LVFFAEDVGSNK) is shared across multiple APP-derived species, including C99, C83, P3 and full-length APP, reducing its specificity for Aβ. APP levels were independently measured using N-terminal and central region peptides, and these measures were not correlated with Aβ, confirming that the SRM-derived Aβ signal was not confounded by full-length APP. However, the assay does not distinguish between Aβ aggregation states or individual monomeric species (Aβ42 or 40). Building on this foundation, the current study employs state-of-the-art highly specific immunoassays^9-11^ to investigate soluble Aβ species in individuals without insoluble Aβ deposition. By quantifying Aβ37, Aβ40 and Aβ42 monomers, we aim to elucidate their relationships with cognitive outcomes and vascular pathologies, particularly CAA. Due to the highly specific assays, we could quantify different Aβ monomers with distinct aggregation propensities^12^ and their correlation to CAA. This work seeks to clarify whether specific Aβ species or their ratios (e.g. Aβ42/40 and Aβ37/42) contribute to disease processes in the absence of Aβ deposition and whether these soluble monomers represent promising biomarkers or therapeutic targets.

## Materials and methods

### Study participants

Participants were drawn from two ongoing community-based clinicopathologic cohort studies on aging: the Religious Orders Study and the Rush Memory and Aging Project (ROSMAP).^13^ Both studies enrolled older individuals without known dementia. Participants underwent annual clinical and neuropsychological evaluations and agreed to brain donation upon death. Follow-up rates amongst survivors exceeded 90%, with an autopsy rate of ∼85%. Mean follow-up 7.5 (SD = 5.5) years. The two studies utilize a large, shared core of testing batteries and are conducted by the same team of investigators, enabling combined analyses; 22.3% of the study participants (*N* = 42) were APOE ɛ2 carriers. Both studies were approved by the Institutional Review Board of Rush University Medical Center and adhered to the Declaration of Helsinki and its amendments. Written informed and repository consents and an Anatomical Gift Act form were obtained from all participants. The current study is restricted to participants without parenchymal Aβ deposition at neuropathologic evaluation, as determined by immunohistochemistry of eight brain regions.^14^

### Cognitive assessment and clinical diagnosis

Cognitive and neurological evaluations were conducted at baseline and annual follow-ups. Twenty-one cognitive performance was assessed, with 19 summarized across five domains: episodic memory (seven tests), semantic memory (three tests), working memory (three tests), perceptual speed (four tests) and visuospatial ability (two tests). Raw scores for each test were standardized using baseline means and standard deviations (SDs) of the entire cohort. A global cognitive score was calculated by averaging the standardized scores across all 19 tests, with domain-specific scores determined similarly.^15^ Higher scores indicate better cognitive function.

### Neuropathologic evaluation

At autopsy, brains were removed and cut into 1-cm coronal slabs. The hemisphere with greater gross pathology was fixed in 4% paraformaldehyde for diagnostic evaluation. Standardized neuropathologic assessments quantified common neurodegenerative pathologies [AD, Lewy bodies, limbic-predominant age-related TDP-43 encephalopathy (LATE) and hippocampal sclerosis (HS)] and cerebrovascular pathologies (cerebral infarcts, CAA, atherosclerosis and arteriolosclerosis), using established protocols.^16^ Neuropathologic raters were blinded to clinical and cognitive data. Neuritic plaques were evaluated in five regions (midfrontal, midtemporal, inferior parietal, entorhinal cortices and hippocampus) using a modified Bielschowsky silver stain. Separately, cortical Aβ burden was assessed immunohistochemically in eight regions (anterior cingulate, inferior temporal, midfrontal, superior frontal, calcarine, entorhinal cortices, hippocampus and angular gyrus) using one of three monoclonal antibodies: 4G8 (1:9000; Covance), 6F/3D (1:50; Dako) or 10D5 (1:600; Elan), as demonstrated in Supplementary Fig. 1 for 4G8 positive staining of CAA. Aβ quantification (percent area Aβ-positive) was performed manually prior to 2019 and transitioned to whole-slide digital scanning and automated image analysis thereafter.^17^

CAA was assessed in four cortical regions (midfrontal, midtemporal, parietal and calcarine).^18^ In each region, meningeal and parenchymal vessels were scored for amyloid deposition using 4G8, from 0 (none) to 4 (circumferential deposition on >75% of vessels). For each region, the higher of the meningeal versus parenchymal score was taken, scores were averaged across regions and the mean was converted to a four-level semiquantitative severity category (none, mild, moderate and severe).

For historical Aβ quantification, regions of interest (midfrontal, superior frontal, cingulate, inferior temporal, entorhinal, angular gyrus, calcarine cortices and hippocampus) were manually outlined (excluding meninges) in Stereo Investigator, followed by systematic random sampling image capture from one section per region. Images were converted from 24-bit colour to 8-bit greyscale, and percent area occupied by Aβ-immunoreactive pixels was computed using Object-Image (v1.62p15).^19^ Beginning in 2019, Aβ-stained sections were scanned on the Aperio AT2 whole-slide brightfield system and Aβ load was computed using the Aperio Positive Pixel Count algorithm as (positive pixels)/(outlined area), yielding a percent Aβ-positive area per slide.^17^

ROSMAP is an ongoing longitudinal cohort spanning over three decades. In this programme, 4G8 staining began in January 2014; prior to that, 10D5 and 6F/3D were used. These antibodies are widely used and were evaluated in small internal tests prior to transitions. The present analyses do not explicitly model antibody differences; this is acknowledged as a limitation. Also, digital Aβ measures are strongly correlated with historical Aβ measures (*r* = 0.83).^17^

### Brain tissue processing

Frozen brain tissue samples were dissected from the dorsolateral prefrontal cortex, specifically Brodmann areas 9 (BA9) and 46 (BA46). The tissue was homogenized in pre-chilled Tris-buffered saline (TBS), supplemented with protease inhibitors, using a Closed System Disposable Micro Tissue Homogenizer (DWK Life Sciences, #1215D61). Homogenization was performed thoroughly to ensure complete tissue disruption. The homogenates were centrifuged at 14 000 × *g* for 30 min at 4°C, and the resulting supernatant was collected and stored in new tubes for downstream immunoassay analysis.

### Aβ immunoassay

Post-cleared brain homogenates were diluted in a wash buffer containing 1% bovine serum albumin (BSA) prepared in TBS with 0.05% Tween-20. Aβ species (Aβ37, Aβ40 and Aβ42) were detected using a 96-well multi-array plate (Meso Scale Discovery, #L15XA-3). Each well was coated with 30 µL of PBS containing 3 µg/mL m266 capture antibody (specific to the mid-region of soluble Aβ; gift from P. Seubert, Elan, plc) and incubated overnight at room temperature. Detection antibody solutions included biotinylated monoclonal antibodies targeting the C-terminal residues of specific Aβ isoforms (clone D2A6H for 37, D8Q7I for 40 and D3E10 for 42[Cell Signalling Technology]), combined with 100 ng/mL Streptavidin Sulfo-TAG (Meso Scale Discovery, #R32AD-5) and 1% BSA diluted in wash buffer. Details on Aβ quantification using SRM were previously described.^7^ Assay sensitivity in plaque-free brain tissue was sufficient for quantifying Aβ37, Aβ40 and Aβ42, but not for reliably detecting lower-abundance species such as Aβ38 or Aβ43. Therefore, these forms were not included in the present analyses. These immunoassays have been previously validated for analytical specificity and reproducibility in cerebrospinal fluid (CSF) and brain tissue,^11^ where the Aβ37/42 ratio was identified as a clinically relevant biomarker.

### Statistical analysis

Characteristics of study participants were summarized using standard descriptive statistics. We applied multivariable logistic regression models to examine the associations of soluble Aβ with CAA, where the 4-level semiquantitative measure of CAA was the ordinal outcome. Additional linear or logistical regression models were used to examine the associations of soluble Aβ measures with other neuropathologic indices. To examine the associations of soluble Aβ with cognitive decline, we applied linear mixed effects models with annual composite cognitive scores as continuous longitudinal outcomes. The models included a model term for time in year before death, which estimates the average rate of change in cognition, and an interaction term between Aβ and time, which estimates additional change with every 1 unit higher in the Aβ level.

All the models were adjusted for age at death, sex and education. The statistical analyses were conducted using SAS software (version 9.4, SAS Institute Inc, Cary, NC) on a Linux platform. Unless otherwise specified, statistical significance was determined at a nominal *α* level of 0.05.

## Results

### Demographic and pathological characteristics of study participants

The primary analyses included brains from 192 individuals without parenchymal Aβ deposition identified by immunohistochemistry. The mean (SD) age at death was 88.1 (7.1) years and the mean (SD) years of education was 16.4 (3.6). Of the participants, 117 were women (61%), and the majority (183 individuals, 95.3%) identified as non-Latino white. Demographic, clinical and neuropathological characteristics are summarized in Table 1. As expected, none of the participants met the pathological criteria for AD based on NIA-AA criteria. Likewise, these brains were less affected by paired helical filament (PHF) tau tangles, TDP-43 pathology or CAA compared to typical AD cases, as shown in Table 1. In our prior work,^7^ we demonstrated that total Aβ burden quantified *via* SRM proteomics correlated with cognitive decline in the absence of Aβ deposition. Expanding on this, we analysed a larger sample of 192 brains (148 in our previous study) using highly specific immunoassays^11^ to measure Aβ37, Aβ40 and Aβ42 in the soluble fractions of cortical brain tissues. This allowed us to investigate correlations between soluble Aβ species and cognitive decline, as well as their association with amyloid-related pathologies, including CAA.

**Table 1:** Characteristics of study participants (*N* = 192)

	Mean (SD) or *N* (%)
Age	88.1 (7.1)
Female	117 (61%)
Education	16.4 (3.6)
Non-Latino white individuals	183 (95.3%)
*APOE* ε4	11 (5.9%)
*APOE* ε2	42 (22.3%)
Clinical diagnosis	
Non-cognitively impaired	92 (47.9%)
Mild-cognitively impaired	52 (27.1%)
Dementia	48 (25.0%)
PHF tau tangle density	0.70 (0.36)
Lewy bodies	44 (23.0%)
LATE (limbic and beyond)	36 (19.0%)
Hippocampal sclerosis	12 (6.3%)
Gross infarcts	63 (32.8%)
Microinfarcts	53 (27.6%)
Cerebral amyloid angiopathy	16 (8.6%)
Atherosclerosis	67 (34.9%)
Arteriolosclerosis	63 (33.2%)
log2 Aβ ratio (37/42)	−2.42 (1.41)
log2 Aβ ratio (42/40)	−1.22 (1.41)
log2 Aβ (SRM)	−5.98 (1.80)
log2 Aβ total (pg/mL)	8.03 (1.36)

### Aβs and Aβ ratios measured by immunoassays do not correlate with cognitive decline

As expected, Aβ levels quantified by SRM were significantly correlated with Aβ measures by immunoassays. Specifically, we observed positive correlations of SRM-quantified total Aβ with total Aβ concentrations by immunoassays (Pearson *r* = 0.26, *P* = 0.001) as well as Aβ42/40 ratio (Pearson *r* = 0.25, *P* = 0.002), and an inverse correlation with Aβ37/40 ratio (Pearson *r* = −0.25, *P* = 0.002).

Aβ levels quantified by SRM remained significantly associated with global cognitive outcomes—both baseline performance (level) and rate of decline (slope)—as well as with decline in visuospatial ability and clinical diagnosis (Tables 2 and 3), consistent with our earlier findings. By contrast, total Aβ concentrations and Aβ ratios measured by specific immunoassays were not associated with cognitive decline. Specifically, neither the total concentration of soluble Aβ (i.e. the sum of Aβ37, Aβ40 and Aβ42) nor the ratios of Aβ42/40 or Aβ37/42 were significantly related to cognitive performance. The only exception was an association between the Aβ42/40 ratio and faster decline in working memory (Table 2). These results suggest that soluble Aβ monomers, as detected by immunoassays, may not strongly contribute to cognitive impairments.

**Table 2:** Quantification of specific Aβs did not correlate with cognitive outcomes (global and domains)

	log2 Aβ (SRM)	log2 Aβ37 pg/mg	log2 Aβ40 pg/mg	log2 Aβ42 pg/mg	log2 Aβ total pg/mg	log2 Aβ ratio (37/42)	log2 Aβ ratio (42/40)
Global							
Level	−0.105 (0.042), 0.013	−0.140 (0.076), 0.067	−0.102 (0.078), 0.193	−0.034 (0.034), 0.321	−0.065 (0.049), 0.186	0.011 (0.046), 0.804	−0.029 (0.049), 0.550
Slope	−0.016 (0.005), <0.001	−0.012 (0.008), 0.115	−0.011 (0.008), 0.164	−0.005 (0.003), 0.111	−0.009 (0.005), 0.064	0.006 (0.005), 0.225	−0.007 (0.005), 0.172
Episodic *M*							
Level	−0.095 (0.050), 0.058	−0.157 (0.089), 0.079	−0.114 (0.092), 0.211	−0.034 (0.040), 0.388	−0.076 (0.058), 0.189	0.005 (0.053), 0.924	−0.023 (0.056), 0.680
Slope	−0.008 (0.005), 0.096	−0.015 (0.008), 0.063	−0.014 (0.008), 0.084	−0.005 (0.004), 0.168	−0.010 (0.005), 0.050	0.004 (0.005), 0.464	−0.004 (0.005), 0.411
Semantic *M*							
Level	−0.014 (0.044), 0.757	−0.126 (0.082), 0.124	−0.113 (0.084), 0.180	−0.024 (0.037), 0.521	−0.055 (0.053), 0.305	−0.002 (0.049), 0.962	−0.004 (0.052), 0.942
Slope	−0.005 (0.004), 0.210	−0.008 (0.007), 0.294	−0.010 (0.008), 0.191	−0.004 (0.003), 0.306	−0.007 (0.005), 0.185	0.003 (0.005), 0.448	−0.003 (0.005), 0.541
Working *M*							
Level	−0.084 (0.044), 0.057	0.061 (0.080), 0.446	−0.035 (0.082), 0.670	−0.016 (0.036), 0.659	−0.020 (0.052). 0.703	0.006 (0.047), 0.895	−0.018 (0.051), 0.721
Slope	−0.009 (0.005), 0.065	−0.007 (0.008), 0.344	−0.005 (0.008), 0.545	−0.006 (0.003), 0.085	−0.008 (0.005), 0.148	0.008 (0.005), 0.077	−0.010 (0.005), 0.043
Perceptual *S*							
Level	−0.034 (0.056), 0.540	−0.068 (0.096), 0.483	−0.012 (0.100), 0.909	−0.001 (0.043), 0.973	−0.017 (0.062). 0.784	−0.021 (0.056), 0.702	0.002 (0.059), 0.969
Slope	−0.006 (0.005), 0.188	−0.007 (0.008), 0.348	−0.004 (0.008), 0.635	−0.003 (0.004), 0.403	−0.005 (0.005), 0.311	0.003 (0.005), 0.573	−0.004 (0.005), 0.395
Visuospatial							
Level	−0.074 (0.044), 0.090	−0.173 (0.079), 0.027	−0.079 (0.081), 0.330	−0.009 (0.035), 0.793	−0.032 (0.052), 0.539	−0.043 (0.047), 0.364	0.011 (0.050), 0.826
Slope	−0.012 (0.005), 0.012	−0.004 (0.008), 0.623	−0.004 (0.009), 0.674	−0.001 (0.004), 0.822	−0.003 (0.006), 0.650	0.0002 (0.005), 0.972	−0.0003 (0.005), 0.963

Linear mixed effects models, statistics are point estimate (SE), *P-*value and all models are adjusted for age, sex and education.

**Table 3:** Association of Aβ quantity and Aβ ratios with CAA in the absence of Aβ deposits

	log2 Aβ (SRM)	log2 Aβ37 pg/mg	log2 Aβ40 pg/mg	log2 Aβ42 pg/mg	log2 Aβ total pg/mg	log2 Aβ ratio (37/42)	log2 Aβ ratio (42/40)
Clinical diagnosis a	0.176 (0.090), 0.050	0.020 (0.163), 0.215	0.121 (0.165), 0.461	0.011 (0.069), 0.871	0.043 (0.100), 0.672	0.051 (0.094), 0.585	−0.021 (0.098), 0.831
PHFtau tangle density b	0.018 (0.015), 0.238	0.033 (0.030), 0.279	0.028 (0.031), 0.359	0.003 (0.013), 0.838	0.013 (0.019), 0.504	0.006 (0.017), 0.725	−0.005 (0.018), 0.797
Lewy bodies a	0.103 (0.108), 0.338	0.185 (0.199), 0.353	0.195 (0.201), 0.333	0.041 (0.087), 0.637	0.100 (0.122), 0.412	−0.010 (0.119), 0.932	0.010 (0.125), 0.936
LATE a	0.192 (0.092), 0.038	0.064 (0.174), 0.708	0.140 (0.171), 0.413	0.034 (0.072), 0.637	0.046 (0.104), 0.657	−0.042 (0.098), 0.669	0.019 (0.103), 0.856
HS a	0.259 (0.150), 0.083	0.021 (0.323), 0.947	0.047 (0.310), 0.879	0.031 (0.134), 0.815	0.025 (0.194), 0.899	−0.053 (0.186), 0.776	0.048 (0.197), 0.807
Gross infarcts a	0.013 (0.101), 0.901	−0.426 (0.204), 0.037	−0.264 (0.198), 0.183	−0.063 (0.082), 0.446	−0.116 (0.122), 0.345	−0.021 (0.104), 0.837	−0.030 (0.112), 0.788
Microinfarcts a	0.116 (0.113), 0.305	−0.264 (0.209), 0.207	0.044 (0.195), 0.821	−0.037 (0.087), 0.672	−0.034 (0.125), 0.788	−0.019 (0.112), 0.866	−0.092 (0.125), 0.464
CAA a	0.292 (0.093), 0.002	0.906 (0.189), <0.001	0.798 (0.189), <0.001	0.379 (0.079), <0.001	0.537 (0.115), <0.001	−0.360 (0.101), <0.001	0.446 (0.108), <0.001
Atherosclerosis a	0.025 (0.087), 0.773	−0.146 (0.161), 0.365	−0.317 (0.166), 0.056	−0.036 (0.070), 0.605	−0.140 (0.102), 0.170	0.015 (0.092), 0.874	0.045 (0.096), 0.639
Arteriolosclerosis a	0.048 (0.087), 0.581	0.059 (0.157), 0.707	−0.185 (0.161), 0.250	−0.021 (0.069), 0.761	−0.104 (0.102), 0.308	0.061 (0.093), 0.510	0.033 (0.098), 0.733

^a^Logistic regression models, statistics are log odd ratio/Log OR (SE), *P*-value.

^b^Linear regression models, statistics are the regression coefficient (SE), *P*-value; and all models are adjusted for age, sex and education.

### Soluble Aβ levels correlate with CAA

In contrast to cognitive decline, total soluble Aβ levels strongly correlated with CAA. Both SRM-quantified total Aβ (Log OR = 0.292 ± 0.093, *P* = 0.002) and the combined immunoassay measurements of Aβ37, Aβ40 and Aβ42 (Log OR = 0.537 ± 0.115, *P* < 0.001) were associated with CAA, as summarized in Table 3. Furthermore, the measurement of individual Aβ37 (Log OR = 0.906 ± 0.189, <0.001), Aβ40 (Log OR = 0.798 ± 0.189, <0.001) and Aβ42 (Log OR = 0.379 ± 0.079, <0.001) all demonstrated strong positive correlation with CAA. Regarding other neuropathological conditions, such as PHF tau tangle density, Lewy bodies, LATE, HS, gross infarcts, microinfarcts, atherosclerosis or arteriolosclerosis, only Aβ37 negatively correlated to gross infarcts (Log OR = −0.426 ± 0.204, = 0.037), as demonstrated in Table 3.

### Longer Aβ Species are associated with CAA

**Figure 1:**
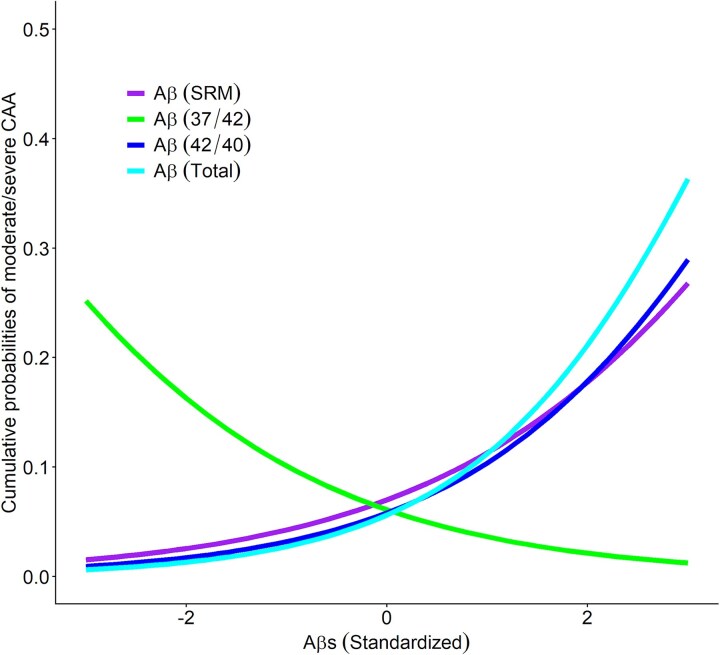
**Correlation between Aβ burden in plaque-free brains and incidence of CAA.** The figure illustrates the associations of individual Aβ measures, i.e. SRM-derived Aβ (red), Aβ37/42 ratio (green), Aβ42/40 ratio (blue) and total Aβ (cyan), with cerebral amyloid angiopathy in older adults without insoluble Aβ parenchymal Aβ deposition (*N* = 192). Each curve represents cumulative probability (*y*-axis) of having moderate or severe cerebral amyloid angiopathy as a function of Aβ level (*x*-axis), estimated from an ordinal logistic regression model adjusted for age, sex and education. We note that Aβ37/42 ratio was associated with less severe CAA (Log odds ratio [Log OR]: −0.360, standard error [SE]: 0.101, Wald chi-square (df = 1): 12.6, *P* < 0.001), SRM derived Aβ (Log OR: 0.292, SE: 0.093, Wald chi-square (df = 1): 9.9, *P* = 0.002), Aβ42/40 ratio (Log OR: 0.446, SE: 0.108, Wald chi-square (df = 1): 17.2, *P* < 0.001) and total Aβ (Log OR: 0.537, SE: 0.115, Wald chi-square (df = 1): 21.9, *P* < 0.001) were associated with more severe CAA.

Although all three Aβ monomers demonstrated a positive correlation with CAA. Further analyses revealed that the Aβ42/40 ratio positively correlated with CAA (Log OR = 0.446 ± 0.108, *P* < 0.001), whilst the Aβ37/42 ratio showed a significant negative correlation (Log OR = −0.360 ± 0.101, *P* < 0.001), as shown in Table 3. These findings suggest that longer Aβ species, particularly Aβ42, may play a critical role in CAA pathogenesis prior to the deposition of parenchymal deposits. No significant correlations were observed between Aβ ratios and other neuropathological conditions. The findings are illustrated in Fig. 1, shows soluble Aβ and Aβ ratios strongly associated with the odds of CAA. Whilst Aβ37 (and Aβ38) can also deposit in vascular amyloid, our prior immunohistochemistry work suggests they may accumulate mainly as surficial layers on pre-existing Aβ42/40 deposits. Thus, the observed negative association of the Aβ37/42 ratio with CAA may reflect a relative protective effect of shorter species against the deposition of longer, more aggregation-prone Aβ forms.

## Discussion

This study builds upon our prior work.^7^ The SRM approach relies on trypsin digestion, which generates peptides derived from the central region of Aβ (LVFFAEDVGSNK) yielding a peptide that is seen in many different Aβ species of varying lengths. Therefore, the SRM approach may have captured contributions from a broader range of Aβ species, including oligomers and longer ‘dark amyloids’ (e.g. Aβ43, Aβ45 and Aβ46).^20,21^ Here, we used highly specific sandwich immunoassays to quantify Aβ37, Aβ40 and Aβ42 monomers in the soluble fraction of cortical tissues. Interestingly, we did not detect strong associations of the total levels of Aβ monomers and their ratios with cognitive decline. This discrepancy likely arises from the technical differences between the methods: whilst immunoassays specifically target monomeric Aβ, SRM indirectly captures aggregated^3,5^ and longer Aβ species^20,21^ that may be more synaptotoxic. On a separate note, the protein extraction methods also differed—SRM required solubilization with 8 M urea, whereas immunoassays used TBS buffer. Although we initially did not expect major differences due to the apparent absence of insoluble amyloid plaques in the tissue, it is possible that 8 M urea partially solubilized subtle plaque-like aggregates that are not detectable histologically. These findings underscore the importance of identifying the specific Aβ species responsible for synaptic toxicity and cognitive decline. Although the pathogenic role of soluble Aβ species remains incompletely defined, our previous work and others^3,6,7,11,22^ demonstrate their clinical and mechanistic relevance. The present findings further support a contribution of soluble Aβ monomers to vascular amyloid pathology, even in the absence of parenchymal plaques.

Despite the lack of clear associations with cognitive outcomes, our findings reveal that soluble Aβ monomer levels in brain tissue are strongly linked to CAA, even stronger than the bulky indiscriminate measurement of the Aβ load with SRM. Total Aβ levels, as well as the Aβ42/40 and Aβ37/42 ratios, significantly correlated with the neuropathological diagnosis of CAA. These results suggest that soluble Aβ monomers may contribute to vascular deposition, consistent with earlier reports that Aβ42 is the initial species deposited in CAA.^23^ Additionally, the opposing correlations of Aβ42/40 and Aβ37/42 with CAA further support the hypothesis that longer Aβ species drive vascular pathology. CAA exists in distinct subtypes, with Type 1 (microvascular) CAA enriched in Aβ42 and Type 2 (larger vessel) CAA enriched in Aβ40. Because our scoring did not differentiate vessel types, we cannot specify whether the associations we observed reflect Type 1 or Type 2 pathology. In addition, although Aβ37 and Aβ38 can deposit in CAA, they appear to do so as secondary or surficial layers on top of longer Aβ deposits rather than initiating pathology. This may explain the negative correlation between Aβ37/42 and CAA severity in our cohort, consistent with the view that enrichment of longer Aβ species is the primary driver of vascular amyloidosis. Amongst other non-AD pathologies, Aβ measurements were not correlated to atherosclerosis or arteriolosclerosis, which supports the hypothesis that CAA-related neurodegeneration and vessel amyloidosis are independent from elevated systemic vascular risk, as previously reported.^24^ These findings have important implications for therapeutic development. Gamma-secretase modulators^9,10,25-27^ that shift Aβ production from longer species (e.g. Aβ42 and Aβ40) to shorter species (e.g. Aβ37 and Aβ38) may prove beneficial in early CAA. Aβ37 and Aβ38 have lower aggregation propensities, potentially mitigating fibril formation and vascular deposition. Notably, 22.3% of the study participants (*N* = 42) were APOE ε2 carriers, whilst we did not find any correlation between APOE genotype and the Aβ monomer measures.

This study has many strengths. The studies have extraordinarily high follow-up and autopsy rates limiting bias due to loss. The participants are community-dwelling and tested in the community-reducing referral bias to tertiary care clinics. The assessment for Aβ entailed several regions and 2–8 sections *per* region on 20 μm sections ensuring that minimal, if any, deposited Aβ was missed. Finally, the sample size of nearly 200 persons without Aβ is large. Weaknesses included low power for smaller effect sizes, a lack of diversity and high age and education. Technically, this study has two main limitations: (i) the use of multiple antibodies for Aβwas not incorporated into the analyses of amyloid pathology and (ii) we were unable to reliably measure Aβ38 or Aβ43 in plaque-free brains due to assay sensitivity constraints, which limits the generalizability of our findings to all soluble Aβ forms.

## Supplementary Material

fcag051_Supplementary_Data

## Data Availability

Data used in this study can be requested through the Rush Alzheimer’s Disease Center Research Resource Sharing Hub (http://www.radc.rush.edu).
