# Supplementary material for: Association of Aβ monomers with cerebral amyloid angiopathy in brains without parenchymal Aβ deposition
Source: Brain Commun. 2026 Feb 19;8(2):fcag051. doi: 10.1093/braincomms/fcag051 (PMC12954484; doi:10.1093/braincomms/fcag051)
Supplement: fcag051_Supplementary_Data [file fcag051_supplementary_data.pdf]

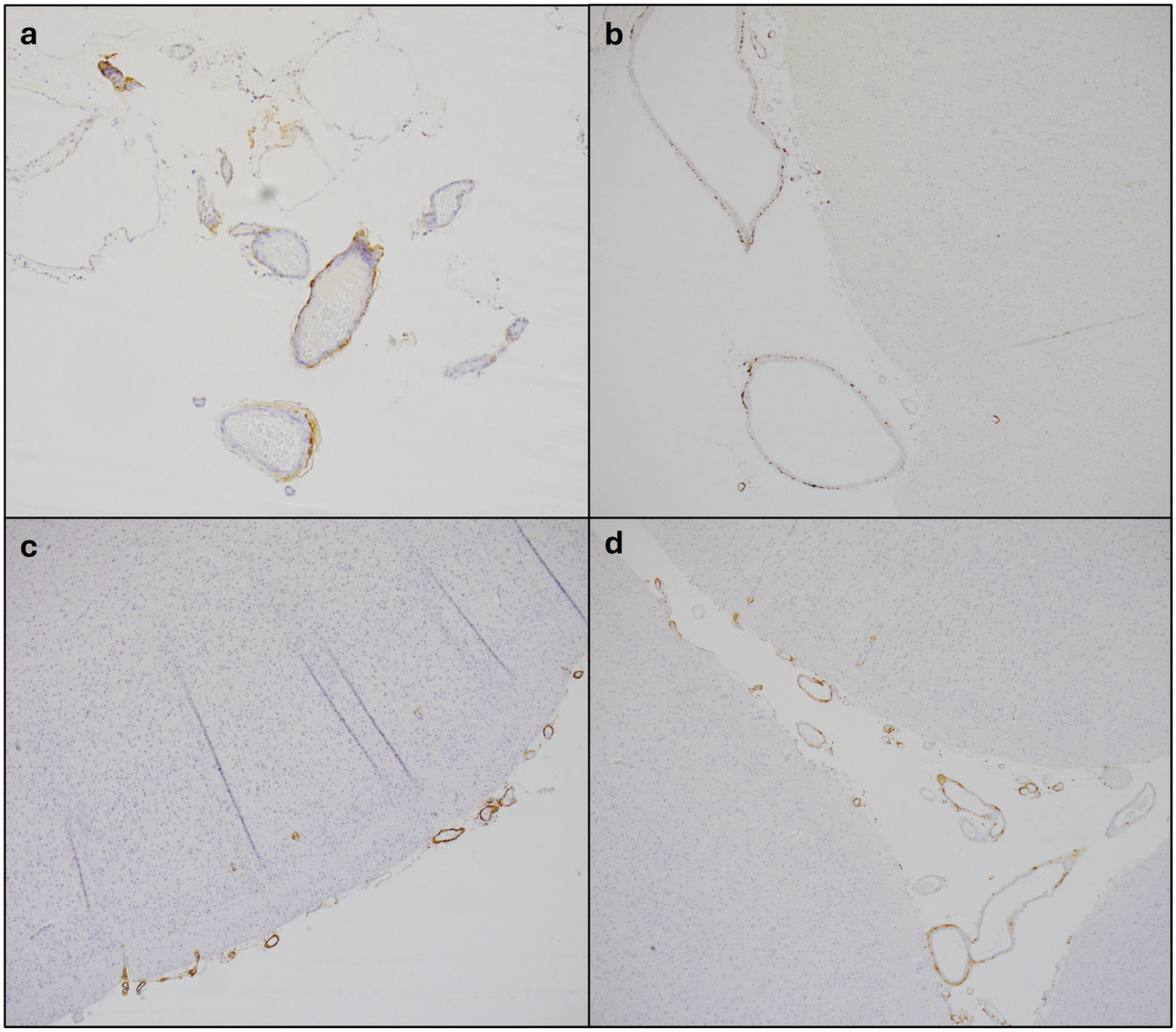

Supplementary figure 1. Representative 4G8-immunostained histological sections of the midfrontal gyrus from four older participants. (a-d), each demonstrating moderate-to-severe CAA pathology in the absence of parenchymal beta-amyloid deposits.
